# Co-Design for Developing and Integrating a Model of Care for Pain Management Centers: Protocol for a Biphase Qualitative Study

**DOI:** 10.2196/59126

**Published:** 2025-06-18

**Authors:** Emma Zhao, Lisa Vaccaro, Yi-Ching Lee, Timothy James Brake, Anastasia Serafimovska

**Affiliations:** 1 Pain Management Centre Royal Prince Alfred Hospital Camperdown Australia; 2 Pain Medicine Research Chris O’Brien Lifehouse Camperdown, New South Wales Australia; 3 Faculty of Medicine and Health The University of Sydney Sydney Australia; 4 Healthy Brain Ageing Program, Brain and Mind Centre The University of Sydney Sydney, NSW Australia; 5 School of Psychology Faculty of Science The University of Sydney Sydney, NSW Australia; 6 Charles Perkins Centre The University of Sydney Sydney, NSW Australia

**Keywords:** chronic pain management, model of care, co-design, clinical care, psychosocial support, quality of life, treatment, medication, Australia, qualitative study, focus group

## Abstract

**Background:**

Current gold standard chronic pain management applies a biopsychosocial lens to clinical care, integrating medication, psychosocial support, and physical reconditioning to promote sustained treatment success, increase quality of life, and control symptoms. However, only 40% of patients with chronic pain report adequate pain management. Unfortunately, evidence describing implemented treatment pathways or models of care (MoCs) that consistently use this holistic approach is lacking.

**Objective:**

The aim of this study is to identify the barriers and facilitators to access and engagement in existing MoCs for chronic pain, explore aspects of the delivery of care that can be improved, and develop an improved MoC for pain clinics in New South Wales by directly liaising with people with chronic pain and their families.

**Methods:**

A 2-phase qualitative study will be conducted using semistructured focus groups. Both phases will follow the same structure, so patients, carers, and clinicians will independently provide input into the proposed MoC, which will then be summarized and integrated by the research team. Participants will be encouraged to interact and speak freely across three core domains: (1) experience of chronic pain management, (2) barriers and facilitators to delivering/accessing a gold standard MoC, and (3) key improvement points to existing models. Each focus group will last 90 minutes and be audio-recorded and transcribed verbatim for qualitative analysis. The focus groups in phase one will generate initial recommendations for primary changes to the existing MoC, and these will then be integrated and presented for evaluation, feasibility, and acceptability by stakeholder groups during phase two.

**Results:**

Data collection commenced in September 2023 and is expected to end by September 2025. As of February 2024, we have completed the focus group with clinicians (n=12) and patients (n=7) in phase one. Data have not been analyzed formally and will be reported in a future publication.

**Conclusions:**

Through the exploration of key stakeholders’ perspectives of the barriers preventing access and the delivery of care in the current MoC for chronic pain, we aim to co-design an appropriate, feasible, and acceptable pathway to be implemented in pain services in the future.

**International Registered Report Identifier (IRRID):**

DERR1-10.2196/59126

## Introduction

Worldwide, chronic pain affects 20%-50% of people and has become a leading cause of disability [1,2]. In 2018, chronic pain was estimated to affect 3.24 million people Australia-wide, and this is estimated to increase to 5.23 million by 2050 [3]. Clinical management of chronic pain applies a biopsychosocial lens [4]. This approach incorporates medication, psychosocial support, and physical reconditioning to promote treatment success [5]. Data from an extensive cross-sectional study reported that only 40% of patients with chronic pain consider their condition to be managed adequately [6]. This is possibly due to the lack of literature demonstrating the implementation of evidence-based treatment pathways or models of care (MoCs) consistently using this holistic approach [2,6].

Pain management centers (PMCs) aim to improve care delivery by promoting a multidisciplinary service for patients with chronic pain, in line with their various needs. This approach promotes collaboration among medical specialists following research-based biopsychological service delivery [7]. However, in Australia, long-standing systemic and site-specific difficulties have impacted the implementation of MoCs within PMCs. These issues include inconsistency (lack of fidelity) in the application of a biopsychosocial pain management approach, untimely (long wait times and poor responsiveness) access to services, minimal capacity to respond to increasing demands for chronic pain management, and insufficiency in identifying patients who require different therapies and facilitating their treatment [7].

In New South Wales (NSW), the MoCs in each local health district vary in accordance with the sociodemographic context and are influenced by the local history of the specific service development [7]. This is reflected in the inconsistency across services, referral pathways into the PMCs, and the provision of outpatient support. This leads to confusion for patients and referrers on how to access pain management services as well as what to expect from these services.

To standardize this approach, the NSW Agency for Clinical Innovation has recommended that pain clinics in all local health districts develop their MoC by adhering to evidence-based principles [7,8]. These include standardized guidelines and treatment/service protocols and pathways, comprehensive screening and assessment to identify the proper level of care, provision of appropriate interventions and therapies, clear definitions of triage and discharge criteria, exploration of technology-based care and interventions, better classification and coding of pain-related presentations, and adequate measurement of patient-reported outcomes (ie, at 3 months, 6 months, and 12 months) [7]. However, these guidelines are not operationalized by any PMC or not consistently applied, nor have patients been directly involved in guiding the development of a MoC that is accessible, appropriate, and feasible for them.

Co-design strategies have been widely used in clinical research, including in the field of chronic pain, to generate user-centric ideas and then successfully implement these ideas in practice [9]. The co-design process usually involves a structured discussion between relevant key stakeholders, including end users (patients and carers), researchers, funding representatives, and policy and decision makers [9]. Crucially, this involvement of multiple key stakeholders (eg, end users) aims to benefit the effectiveness of health-related interventions and services [9]. In addition, the co-designed approach aims to produce services that are more acceptable and applicable to these groups. Engaging stakeholders in service redesign enables longer-term refinement and sustainability of resources, and can inform the governance and policy required to optimize service structure and funding into the future [10].

This study explores the barriers and facilitators to implementing evidence-based, biopsychosocial MoCs from the perspectives of key stakeholders without aiming to measure individual adherence. Through focus groups with patients, carers, clinicians, and clinical leaders, we aim to identify challenges in current MoCs and codevelop practical recommendations. These stakeholder-informed insights will guide the future design and potential piloting of a more responsive, integrated MoC for PMCs.

## Methods

### Study Design

The project will be a 2-phase (sequential) study.

#### Phase One

Phase one will include semistructured focus groups of individual stakeholders (patients, clinicians/leaders, carers, and families). It will aim to identify the initial barriers and facilitators within the existing MoC at Royal Prince Alfred Hospital (RPAH).

#### Phase Two

**Figure 1:**
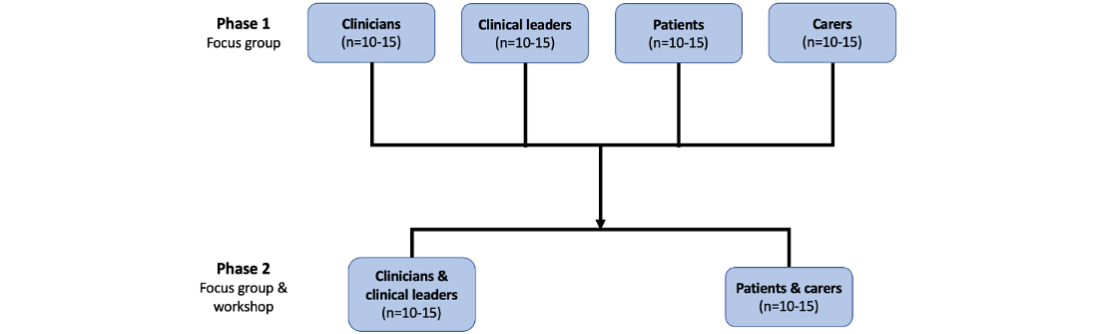
Visual representation of a biphase sequential focus group study, outlining each stakeholder group per phase.

Phase two will involve 2 half-day workshops (2 hours) and focus groups (1 hour) held on-site at RPAH. Each of the combined stakeholder groups (clinicians and clinical leaders, and patients and caregivers) will have one workshop, as shown in Figure 1. The workshop will explain the current MoC again and discuss more detailed study findings from phase one, with potential areas for improvement identified. The focus groups will examine the MoC and elaborate on any improvement across key elements (eg, design, service provision).

### Study Setting

The focus groups will be run from the RPAH in the available board and meeting rooms or on Zoom (Zoom Communications) or Microsoft Teams.

### Participants

Overall, we anticipate 4-6 focus groups with 5-10 participants in each group (see Figure 1). While we recognize the variability in pain types, treatment pathways, and professional roles, this heterogeneity reflects the real-world complexity of pain care and is essential for developing a flexible, contextually adaptable model. This is based on previous research [11] suggesting 3-6 focus groups are required to achieve thematic/code saturation with a semistructured interview guide, which is supported by evidence on meaning saturation for identifying diverse stakeholder perspectives [12].

We use a purposive (nonrandom) sampling strategy to recruit participants with relevant experience in pain care and education, aiming to obtain “information-rich” cases that can inform service improvements [13]. The credibility of this approach is boosted by including representatives from key stakeholder groups in our research [14].

Stakeholder groups consist of patients who have engaged in multidisciplinary care (eg, at least one psychology and one pain specialist consultation), carers who are directly involved in the care of these patients, clinicians actively involved in multidisciplinary pain care at RPAH or other sites, and external bodies (ie, government and nongovernment organizations engaged in policy or guidelines related to pain management on a district, state, or national scale).

Focus group composition will be structured in 2 phases. Phase one features homogeneous stakeholder-specific groups (eg, patients only, clinicians only) to encourage open discussion in a supportive environment, especially around sensitive topics [15]. This approach also shifts the power balance toward participants, which is ethically important when engaging groups whose voices are often underrepresented in research and policy [16].

Phase two introduces heterogeneity to promote cross-stakeholder dialogue and refine ideas. Specifically, we will group patients and carers (group 1) and clinicians with external stakeholders (group 2), as shown in Table 1. The first phase focuses on idea generation to reduce the influence of power dynamics between patients, carers, and clinicians. This structure reflects the need to balance homogeneity in phase one (to support openness) with diversity in phase two (to capture divergent perspectives) [17-19]. Group sizes of 5-10 per session are consistent with qualitative standards for achieving thematic and meaning saturation [11,12].

**Table 1:** Summary of inclusion and exclusion criteria per stakeholder focus group.

Group	Inclusion criteria/definition	Exclusion
Patients (n=5-10)	Patients from RPAH’s^a^ Pain Management Centre who are currently receiving or have received treatment in the past for chronic pain (at least one initial session with a pain specialist and pain psychologist)	Patients who decline to participate or are ineligible given competing health priorities or general difficulties (eg, cognitive problems, self-reported poor English skills)
Carers (n=5-10)	Individuals providing care for patients currently or previously receiving treatment in the Pain Management Centre (eg, family members, friends, professional support workers)	Participants who decline to participate
Clinicians (n=5-10)	Directly involved in multidisciplinary clinical care for patients within their professional capacity at RPAH or other hospitals (eg, physiotherapists, psychiatrists, occupational therapists)	Participants who decline to participate or are ineligible because they have no professional experience or training related to chronic pain or model of care implementation
Clinical leaders (n=5-10)	Managers, service planners, patient navigators, and pain association leaders at professional bodies (eg, Agency for Clinical Innovation) who are responsible for decision-making at institutional or state/national levels	Same as for clinicians

^a^RPAH: Royal Prince Alfred Hospital.

### Data Collection

#### Focus Group

The focus groups will be used for data collection in accordance with the predetermined open-ended focus group guide. The focus group questions are developed to discuss current chronic pain management, barriers and facilitators for the delivery of the current MoC, and future avenues of improvement.

Focus groups (phases one and two) will be conducted face-to-face or via Microsoft Teams. They will take 60 minutes and be facilitated by a research project staff member and one of our study investigators. Each focus group will be digitally recorded for video/audio (the anticipated duration is 60 minutes) and transcribed verbatim by the research team. In addition, a separate researcher from the team will observe each group and take notes on points of particular agreement or disagreement to ensure accuracy.

Facilitators will engage in ongoing critical reflection before and after interviews and focus groups to support reflexivity and reduce bias, including maintaining reflexive field notes. Where possible, interviews and groups will be conducted by researchers not directly involved in participants’ clinical care. The research team will discuss and document positionality and potential power dynamics in team meetings throughout the study.

#### Survey

In phases one and two, all participants will be given questionnaires as part of the focus group. The questionnaires will collect the primary demographic data, including gender, age, pain history (patients/carers), and work experience (clinicians and clinical leaders).

Participant (patient) data from the electronic Persistent Pain Outcomes Collaboration (ePPOC) [20] questionnaires will also be extracted. The ePPOC is routinely administered before and after treatment for patients attending multidisciplinary pain clinics across Australia and New Zealand, including the RPAH PMC. Information extracted from the ePPOC will be deidentified and include information related to pain, physical disability, medications, cognition, mood, and health care use. ePPOC does not collect data from carers or clinicians; therefore, the custom tool complements, rather than overlaps with, ePPOC and allows us to characterize all stakeholder groups involved in the study.

### Recruitment

Potential participants for phases one and two will be recruited separately. Participants from phase one may also consent to participate in phase two; however, consent will be collected for participation in each phase separately.

#### Clinicians and Clinical Leaders

For clinicians and clinical leaders, permission will be sought from clinical departments to send out invitations regarding the study, on behalf of the research team. Information will be distributed at departmental, multidisciplinary, and staff meetings to raise awareness and facilitate recruitment. Study staff will then contact those who reply positively to explain the study and what their participation would involve.

#### Patients and Carers

For patients and their carers, an invitation will be given to patients attending pain management outpatient clinics. Research staff will only approach patients or families after agreement is obtained from their treatment team. The research staff will explain the study, check eligibility criteria, and schedule a separate meeting to obtain consent and answer any further questions. This will be at a mutually convenient time and date. Potential participants will have at least 24 hours to consider participation.

#### Snowball Sampling

Those invited to participate (clinicians/patients) will be encouraged to refer their colleagues, patients, or carers to participate. They can either ask permission from their colleagues/patients/carers for the study staff to contact the potential participants and forward their contact details (email or phone) to the study staff (with their permission) or pass on contacts of the study staff to potential participants who might initiate contact with the research team. Once a member of the study team is in connection with the potential participant, they will explain the study, determine if the study is suitable for the potential participant, and forward a copy of the study information to consider before scheduling a meeting (either face-to-face or on Microsoft Teams) at a time and date that is mutually convenient. The purpose of this meeting is to ensure ample time is given to consider the study, to provide opportunities to answer questions, and to finalize consent.

### Data Analysis Plan

#### Focus Group Data

Qualitative interviews and focus group notes will be transcribed verbatim and entered into NVivo version 14 (Lumivero), and two people will independently code the data. Inductive and deductive thematic analysis [21] will be used to identify themes within the data related to the research questions. Each transcript will be deidentified, read closely, and coded line by line inductively and descriptively before being coded deductively based on the agreed set of themes. These will then be integrated into the analysis of subsequent focus groups until meaning saturation is achieved.

#### Survey Data

Descriptive statistics from existing clinical data and a brief demographic questionnaire will be used to describe the demographic characteristics of all stakeholders to understand relevant contextual factors affecting their experience (eg, years experiencing chronic pain, interventions received, years of professional experience). Frequency and percentage will be used to describe these variables. Chi-square comparisons (ie, post hoc, with *P*<.05 considered as significant) will be conducted between groups to identify whether differences in sociodemographic and education characteristics can be captured. All statistical analyses will be performed using SPSS version 26 (IBM Corp) [22].

#### Withdrawal of Consent

If participants decide to withdraw from the study, the research team ceases to collect study information. Participants will be informed that withdrawal is possible at any time, with no consequences for their care or existing relationships with clinicians. If participants choose to withdraw before their initial focus group, their data will be erased from study records. However, their data will be retained and deidentified once they have participated in a focus group. Extracting an individual line of information from a communal focus group is difficult.

### Ethical Considerations

#### Ethics

Ethics approval for phases one and two of this project has been provided by the Ethics Review Committee (RPAH Zone) of the Sydney Local Health District (SLHD; HREC/2023/X23-0224). Ethics approval was granted in early September 2023, and data collection began shortly after.

The study investigators will ensure all study procedures are in accordance with the National Health and Medical Research Council (NHMRC) Statement on Ethical Conduct in Human Research 2007 (updated 2018) [23] and NHMRC Australian Code for the Responsible Conduct of Research [24]. This work was conducted in accordance with the 1964 Declaration of Helsinki. Participants were not financially reimbursed, they were provided with refreshments during and after the focus groups.

#### Informed Consent

All participants provided informed consent and were able to opt out at any time without any repercussions for their care or engagement with the service. Their privacy and confidentiality are retained by appropriate data collection and storage in alignment with approved SLHD and The University of Sydney protocols.

#### Dissemination

It is anticipated that the findings and relevant outcomes of this project will be reported in international peer-reviewed journal articles and presented at relevant national and international conferences. A summary of research findings will be made available to participants.

## Results

Data collection commenced in September 2023 and is expected to end by September 2025. As of February 2024, we have completed the focus group with clinicians (n=12) and patients (n=7) in phase one. Data have not been analyzed formally and will be reported in a future publication. Preliminary analysis from the clinician group suggested that the current MoC needs to be improved after further involvement from stakeholders, such as patients, carers, researchers, funding representatives, and policy and decision makers for future practice.

## Discussion

### Expected Findings

The primary aims of this study are to identify the barriers and facilitators to access and engagement in existing MoCs for chronic pain in PMCs in NSW, including pain education programs. This will include input from clinicians, patients, and carers from pain clinics across NSW; explore aspects of the delivery of care (including accessibility, quality, and feasibility) that can be improved for people with chronic pain; and uniquely co-design an improved MoC for pain clinics in NSW by directly liaising with people with chronic pain and their families.

Current MoCs vary significantly between individual clinics, including in adherence to existing guiding principles [8] and the specific needs required by the populations they serve. Some research about establishing MoCs exists, but most of it is focused explicitly on particular health conditions, such as lower back pain and opioid management [25-27]. Most of these services have evolved organically, and patients currently have minimal, if any, input into the care they receive [28]. This lack of awareness among users of PMCs minimizes their confidence and ability to engage with the services offered directly [7]. At the same time, pain clinicians have few opportunities to systematically evaluate and update the care plans they are providing to adapt to emerging evidence on biopsychosocial support for pain management [29].

The findings of this proposed study could have significant implications for current pain management services, given the rapidly expanding population of people living with chronic pain in Australia and worldwide. An integrated model has been suggested to manage chronic disease, including chronic pain [30]. The core components of managing chronic disease include well-defined care guidelines, an existing health delivery system, and a continuous improvement process within the delivery system [30]. Therefore, within the current established MoC in the Australian health system, the study will explore across stakeholders to provide tight feedback loops and expedited implementation of evidence-based interventions that are tailored to the contextual features of the service, including scope, staff, and other resources. Additionally, a more self-sustaining and iterative MoC can be adapted across time and resources to match population needs and address traditional barriers by promoting tailored yet holistic care in a consistently biopsychosocial way.

Subsequently, it may reduce the cost and burden on existing strained resources and promote adherence to best practices. In current clinical practice, the evaluation of long-term effectiveness within the existing MoC is unclear. In contrast, short-term evidence may not be convincing enough for decision-making and operational management in pain management services [31]. This study will provide a concept of a stratified cost-effectiveness care model and promote a data analysis to evaluate the current pain service in the future.

### Limitations

The primary limitation of this study is that the findings may be highly localized to health services in Australia. However, the process will be flexible enough to be generalized across other centers. Researcher presence (including dual roles across the clinician-researcher space) can potentially influence qualitative findings (across power hierarchies or comfort in content discussed). Another potential limitation is using a custom-developed demographic questionnaire alongside the ePPOC dataset. However, the alignment of the custom tool with validated sources and its role in complementing, rather than duplicating, ePPOC data support the reliability and interpretability of the combined dataset.

The study uses purposive and snowball sampling strategies, which may introduce selection bias by overrepresenting individuals who are more engaged, vocal, or closely connected to clinical services. This may limit the generalizability of findings to the broader population of patients, carers, or clinicians. However, this approach is appropriate for in-depth qualitative exploration and co-design, where capturing rich, experience-based insights from informed stakeholders is critical to developing a feasible and contextually relevant MoC.

A further limitation is the potential for bias introduced by researcher positionality, particularly where facilitators are known to participants or affiliated with the care team. To enhance trustworthiness, reflexive practices will be used throughout data collection and analysis, including reflexive journaling, debriefing sessions, and the involvement of multiple coders during thematic analysis.

### Conclusion

In this protocol, we outline the development of MoCs for PMCs by co-designing with patients, carers, clinicians, and clinical leaders. This research project may help address current barriers to engaging with and delivering chronic pain care. Furthermore, the novelty of the co-designed MoC will provide a new approach to evaluating the care model of the health care system in the future.

## Abbreviations

ePPOC: electronic Persistent Pain Outcomes Collaboration
MoC: model of care
NHMRC: National Health and Medical Research Council
NSW: New South Wales
PMC: pain management center
RPAH: Royal Prince Alfred Hospital

## Data Availability

The datasets generated and analyzed during this study will be available from the corresponding author upon reasonable request.
